# The profiling and analysis of gene expression in rat temporomandibular joint disc tissue and its derived cells

**DOI:** 10.1016/j.jobcr.2025.03.018

**Published:** 2025-04-21

**Authors:** Haruna Kashio, Rie Takai, Ayuko Takada, Yuya Nakao, Nattakarn Hosiriluck, Masahiro Iijima, Yoshihiro Abiko, Itaru Mizoguchi, Toshiya Arakawa

**Affiliations:** a Division of Biochemistry, Department of Oral Biology, School of Dentistry, Health Sciences University of Hokkaido, Japan; b Division of Orthodontics and Dentofacial Orthopedics, Department of Oral Growth and Development, School of Dentistry, Health Sciences University of Hokkaido, Japan; c Department of Masticatory Science, Faculty of Dentistry, Mahidol University, Bangkok, Thailand; d Division of Oral Pathology, Department of Oral Growth and Development, School of Dentistry, Health Sciences University of Hokkaido, Japan; e Division of Orthodontics and Dentofacial Orthopedics, Tohoku University Graduate School of Dentistry, Japan

**Keywords:** Temporomandibular joint disc, Gene expression, Collagen, Proteoglycan

## Abstract

**Objective:**

The temporomandibular joint (TMJ) disc is composed of a fibrocartilaginous connective tissue. Its dysfunction, resulting from excessive jaw movement, can lead to TMJ disorders. In this study, we aimed to investigate the crucial molecular information regarding the extracellular matrix (ECM), which would be necessary for treating such disorders through TMJ disc regeneration. To achieve this, we compared the gene profiles of TMJ disc tissues, their derived cells, and the periodontal ligament (PDL) in our previous study.

**Methods:**

TMJ discs were isolated from male Wistar rats. Cells derived from the TMJ discs were cultured, and mRNA extracted from the disc tissues and derived cells was analyzed for gene profiling via microarray hybridization. Additionally, we compared the ECM expression between the TMJ disc and the PDL.

**Results:**

Collagen (types I, II, III, and VI) and proteoglycans (biglycan and fibromodulin) were highly expressed in the TMJ discs. Significant reduction in decorin, fibromodulin and COL2 were observed in the TMJ-derived cells than in the tissue. Type VI collagen was the third most highly expressed in both the TMJ disc and PDL tissues, following types I and III.

**Conclusions:**

Collagen types VI and II were prominently expressed, followed by collagen types I and III, in TMJ disc tissues, reflecting the unique functions of the disc. Type VI collagen was highly expressed in both TMJ disc and PDL tissues. Overall, type VI collagen might be a key molecule for TMJ disc regeneration, ensuring elasticity and cushioning, and could provide new insights for TMJ regeneration.

## Graphical abstract

**Figure:**
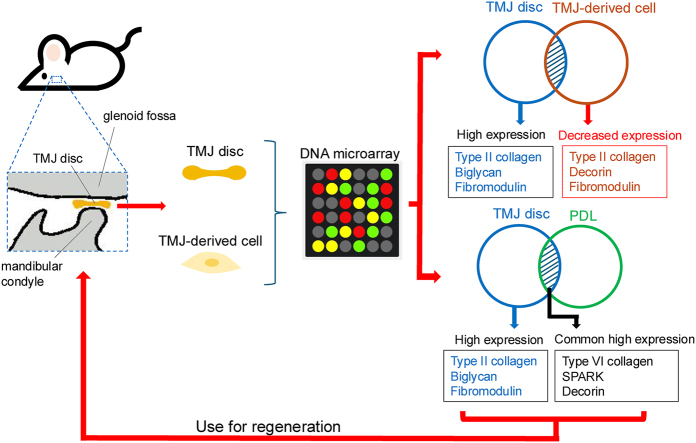

## 1 Introduction

The temporomandibular joint (TMJ) disc, located between the mandibular condyle and the glenoid fossa, is a crucial fibrocartilaginous tissue that absorbs loads to facilitate jaw movements such as chewing and speaking. However, weakening of this disc can result in deformation or displacement, impeding smooth movement, and often leading to perforation or tearing.^1^ When the disc loses its shock-absorbing capacity due to an excessive jaw load, resorption of the mandibular condyle may occur, necessitating surgical reconstruction. Tissue engineering offers a promising approach to disc regeneration,^2^ with fibroblasts serving as valuable resources owing to their relative ease of isolation than that of stem cells.

The mechanical properties of the TMJ disc are intrinsically linked to the extracellular matrix (ECM), and disc degradation may result from modifications in the expression and composition of the ECM.^3^ Several types of collagen (I, II, III, V, VI, and XI), proteoglycans (biglycan and decorin), and non-collagen proteins (fibronectin and elastin) have been identified in the ECM of the TMJ disc^4^^,^^5^; however, which ECM component in TMJ disc-derived cells is the most important for tissue regeneration in order to restore function in the case of TMJ disc disorders remains unclear.

We designed two studies to identify the specific ECM molecules involved in tissue regeneration in TMJ discs. The first study aimed to analyze the ECM specific to TMJ disc tissue by comparing the gene expression between TMJ disc tissue and TMJ disc-derived cells. TMJ disc cells dedifferentiate due to decreased ECM expression during passaging under experimental conditions.^6^ This reduction in ECM expression, resulting from environmental changes in the culture, is considered to affect the properties of the cells. Therefore, understanding the differences in ECM gene expression patterns between TMJ disc tissues and the derived cells would be important for the effective regeneration of TMJ disc tissue using TMJ disc-derived cells. The second study aimed to analyze the molecules related to ECM functions, such as cushioning, in TMJ discs. The TMJ disc functions similar to the periodontal ligament (PDL), which is constantly subjected to significant occlusal forces. Both the TMJ disc and PDL are interposed between hard tissues. Additionally, the PDL and intermediate zones of the TMJ disc are extremely thin tissues. In our previous study, we elucidated the ECM gene profile of the PDL.^7^ Thus, identification of the ECM molecules indispensable for TMJ disc function may be possible by comparing the gene expression between TMJ disc and PDL tissues.

In this study, we identified not only the unique ECM genes and specific molecules for TMJ disc regeneration by comparing the gene expression in TMJ disc tissue and its derived cells but also the indispensable ECM molecules necessary for TMJ disc functions, such as cushioning, by comparing with those of the PDL.

## 2 Materials & methods

### 2.1 Animals

We used 4-week-old rats for our study, since the ECM components in the TMJ discs of juvenile rats are known to be high.^4^ A total of 20 male Wistar rats, each weighing 95 ± 6 g, were procured (Sankyo Labo Service, Inc., Tokyo, Japan) and used. All rats were housed in ventilated, filter-topped cages with a 12-h/12-h light/dark cycle, and provided with autoclaved food and water ad libitum. All animal procedures rigorously adhered to the Animal Research: Reporting of In Vivo Experiments (ARRIVE) guidelines.

### 2.2 Materials

Minimum Essential Medium Eagle-alpha modification (a-MEM), fetal bovine serum (FBS), and all general reagents were purchased from Sigma-Aldrich Co. (St. Louis, MO, USA). Bambanker™ was acquired from Nippon Genetics (Tokyo, Japan) and Block-Ace was acquired from DS Pharma Biomedical Co. (Tokyo, Japan). The primers were supplied by Hokkaido System Science Co., Ltd. (Sapporo, Japan). Antibodies against Col2 and GAPDH were procured from Abcam (Cambridge, UK) while Col6A was from Santa Cruz Biotechnology (Santa Cruz, CA, USA) and beta-actin was from Sigma-Aldrich Co.

### 2.3 Surgical procedures for TMJ disc isolation

Rats were anesthetized using 5 % isoflurane inhalation. They were euthanized by cervical dislocation after anesthesia, and their heads were removed. The TMJ was exposed using scissors, and the discs in contact with the mandibular condyle were carefully separated from the surrounding tissues using fine forceps and a surgical scalpel blade (No. 10) (Appendix Fig. a and b).

### 2.4 Cell isolation and culture from TMJ disc and PDL

Eight TMJ discs were collected from four rats. The TMJ discs were minced into four pieces using a surgical scalpel blade (No. 11), immersed in PBS, and transferred to a collagen-coated Petri dish. They were cultured in α-MEM with 10 % NBS, penicillin (100 units/ml), streptomycin (100 μg/ml), and L-glutamic acid at 37 °C with 5 % CO_2_. At 80 % confluence, the TMJ disc cells were subcultured and frozen with Bambanker™, as a stock, at 1 × 10^5^ cells/ml. Cells were isolated after three passages, and TMJ-derived cells from the fourth to sixth passages were used for the experiments. TMJ-derived cells were cultured in α-MEM with 10 % NBS, penicillin (100 units/ml), streptomycin (100 μg/ml), and L-glutamic acid at 37 °C with 5 % CO_2_. Human PDL fibroblasts harvested from orthodontic treatment extractions served as a positive control for fibroblasts^7^ as shown briefly in Appendix materials and methods.

### 2.5 Microarray analysis and qPCR

Total RNA was extracted from the eight TMJ discs harvested from four rats, and from the TMJ-derived cells, using an RNeasy Mini Kit (Qiagen, Hilden, Germany). Microarray hybridization analysis using the total RNA was performed by Hokkaido System Science Co., Ltd. (SurePrint G3 Rat 8 × 60 K ver.1.0; Agilent Technologies). For qPCR, RNA samples (1 μg) were converted to cDNA with SuperScript™ II Reverse Transcriptase (Thermo Fisher Scientific, MA, USA) and analyzed using specific primers for COL1A1, COL2A1, COL6A2, and beta-actin with the KAPA SYBR® FAST qPCR Master Mix Kit (KAPA BIOSYSTEMS, MA, USA). The primers used are listed in Appendix Table. Gene expression fold change was calculated using the 2^−ΔΔCt^ method.

### 2.6 Western blotting

Western blot analysis for COL2, COL6A2, beta-actin, and GAPDH was conducted for the eight TMJ discs collected from four rats and the derived cells, using rat costal cartilage and human PDL as positive controls. Protein preparations from TMJ disc tissues and cells were obtained using a Polytron homogenizer PT10/35 (KINEMATICA GmbH, Switzerland) using a sample buffer (50 mM Tris-HCl pH 6.8, 1 % SDS, 10 % glycerol, 6 % 2-mercaptoethanol, and BPB). The protein concentration was measured by the bradford method using XL-Bradford (APRO SCIENCE Co, Tokushima, Japan) with GeneQuant Pro spectrophotometry (Amersham, UK). Protein samples (10 μg) were separated by 5–20 % SDS-PAGE and transferred to PVDF membranes. The membranes were blocked overnight with 10 % Block-Ace, and incubated with primary antibodies (Col2: ab188570, Col6A2: sc-374566, GAPDH: ab8245, and beta‐actin: AC‐15) and secondary antibody (Col6A2, GAPDH and beta‐actin: sheep anti-mouse IgG HRP, and Col2: donkey anti-rabbit IgG HRP) (Thermo Fisher Scientific, MA, USA). Protein bands were detected using Enhanced Chemiluminescence (ECL) and a CCD camera system with an ATTO CS Analyzer 3.0 (Tokyo, Japan).

## 3 Results

### 3.1 Microarray analysis of the ECM in TMJ disc tissue and its derived cells

**Fig. 1:**
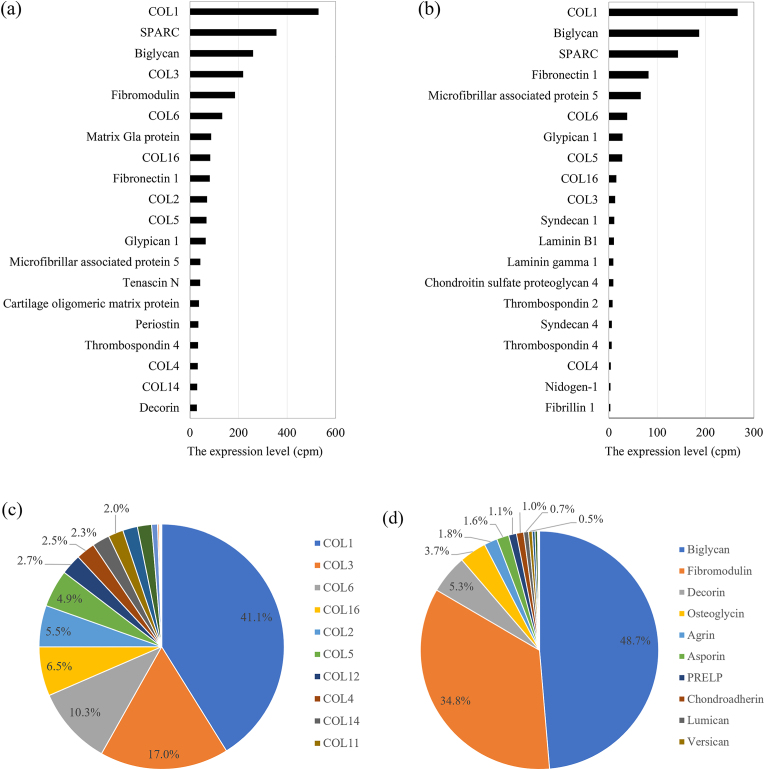
The expression levels of the top 20 ECM genes in the TMJ disc tissue (a) and the TMJ-derived cells (b). Proportion of 27 types of collagen (c) and 20 types of proteoglycan (d) in the TMJ disc tissue.

High expression of COL1, SPARC, and biglycan was observed in both the TMJ disc tissue and the derived cells by microarray analysis, although the levels were higher in the tissue (Fig. 1a and b). The TMJ disc tissue expressed 23 types of collagen and 20 types of proteoglycan, COL1 being the most prevalent, followed by COL3 and COL6 (Fig. 1c). Biglycan was the most abundant proteoglycan, followed by fibromodulin and decorin (Fig. 1d). Significant reduction in decorin, matrix Gla protein, fibromodulin and COL2 were observed in the TMJ-derived cells than in the tissue (Table 1).

**Table 1:** Changes in ECM gene expression in the TMJ disc tissue and the derived cells. This table shows the fold changes in highly expressed ECM genes in the TMJ disc tissue, which are reduced in the derived cells. Gene expression intensity was measured in counts per minute (cpm).

Gene	TMJ disc tissue (cpm)	TMJ disc cells (cpm)	Fold Change
Decorin	28.212	0.004	7818.758
Matrix Gla protein	87.033	0.022	4003.594
Fibromodulin	185.773	0.050	3708.496
COL2	70.231	0.030	2334.988
Cartilage oligomeric matrix protein	36.955	0.052	714.049
COL14	29.571	0.160	185.371
Tenascin N	42.196	2.302	18.331
Periostin	34.753	1.930	18.010
COL3	219.204	12.989	16.877
COL4	32.238	3.572	9.025
Thrombospondin 4	33.438	5.847	5.719
COL16	83.420	15.529	5.372
COL6	133.133	38.084	3.496
SPARC	356.026	143.035	2.489
COL5	67.955	27.738	2.450
Glypican 1	64.575	28.191	2.291
COL1	529.471	266.419	1.987
Biglycan	259.965	186.765	1.392
Fibronectin 1	81.241	82.224	0.988
Microfibrillar associated protein 5	42.528	65.986	0.645

### 3.2 Expression of COL1, COL2, and COL6 in TMJ disc tissue and its derived cells based on qPCR and western blotting

**Fig. 2:**
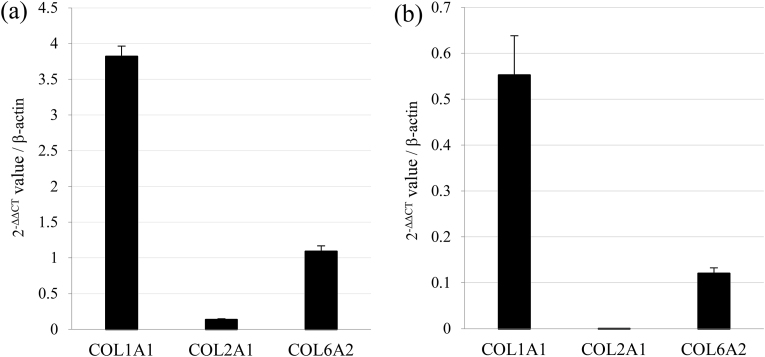
Real-time PCR analysis of COL1A1, COL2A1, and COL6A2. Comparison of gene expression between the TMJ disc tissue and cells, showing the 2^−ΔΔCT^ values in the TMJ disc tissue (a) and the TMJ-derived cells (b).

**Fig. 3:**
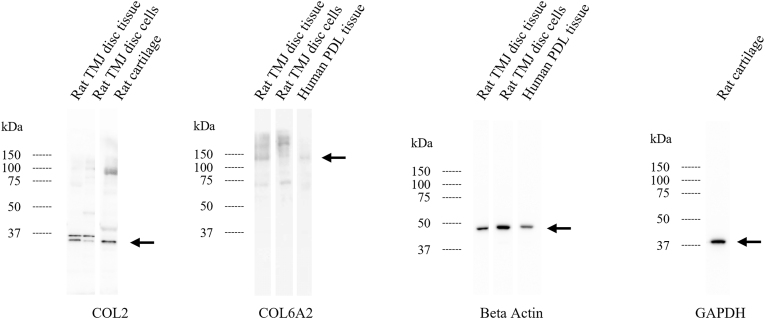
Western blot analysis of COL2A1 and COL6A2 in the TMJ disc tissue and the derived cells. Extracts from the rat cartilage (for COL2) and the human PDL tissue (for COL6A2) were used as positive controls for each blot. Beta-actin served as an internal reference for the TMJ disc tissue, the TMJ-derived cells, and the human PDL tissue. GAPDH was used as an internal reference in the rat cartilage. Arrows indicate the expected protein bands.

The gene and protein expression of COL1, COL2, and COL6, which are important ECM components in the TMJ disc, was analyzed using qPCR and western blotting. Both the levels showed COL1A1 as the most highly expressed, followed by COL6A2 and COL2A1, in the TMJ disc tissue as well as in the derived cells; COL2A1 was expressed only slightly (Table 2, Fig. 2a and b). This pattern was consistent with the microarray results (Fig. 1a and b). Western blotting confirmed the presence of COL2 in both TMJ disc tissue and the derived cells, with reduced expression in the cells, whereas COL6A2 was consistently present in both (Fig. 3). The results were collectively consistent with both microarray and qPCR findings (Table 2, Fig. 1, Fig. 2a, and b).

**Table 2:** C_T_ (Cycle Threshold) mean values measured by quantitative PCR for COL1A, COL2A1, and COL6A2 in the TMJ disc tissue and the derived cells. β-actin was used as an internal control.

	β-actin	COL1A1	COL2A1	COL6A2
TMJ tissue Cт Mean	17.938	15.970	20.797	17.782
TMJ cells Cт Mean	15.689	16.713	33.449	18.904

## 4 Discussion

The distribution of collagen subtypes varies across tissue types. The cellular composition of the TMJ disc includes fibroblasts and fibrochondrocytes, with the latter possessing characteristics of both fibroblasts and chondrocyte cells.^4^ While type II collagen is typically associated with chondrocytes,^5^ our findings revealed high levels of type II collagen, in conjunction with types I and III, in the TMJ disc tissue (Fig. 1, Fig. 2a). In contrast, other tissues, such as the PDL and skin, predominantly contain collagen types I and III, which resist expansion.^5^ Type II collagen, mainly found in the cartilage, aids in resisting compressive forces owing to its water retention capabilities, especially in the intermediate zone of the TMJ disc.^5^ Given its unique cellular composition, the TMJ disc may represent a distinct tissue type, where collagen type II, alongside types I and III, plays a crucial role in maintaining mechanical properties. This suggests that the TMJ disc, with its amalgamation of fibroblast- and chondrocyte-like properties, is a specialized structure in which collagen type II contributes significantly to its mechanical integrity.

In rat TMJ discs, type VI collagen constituted 10.3 % of the total collagen, ranking third after types I (37.3 %) and III (15.4 %) (Fig. 1c). The presence of the COL6A2 subunit was confirmed by qPCR and western blotting (Fig. 2, Fig. 3). Mouse TMJ disc,^4^ cartilage^8^ and PDL tissues^7^ from our previous study showed a similar ranking, with type VI collagen being the third most abundant. Despite the higher type I collagen content in the PDL, the overall collagen composition in the TMJ disc and PDL was comparable, highlighting the prominence of type VI collagen in tissues subjected to significant occlusal forces. Type VI collagen, which is localized in the pericellular regions of fibroblasts and chondrocytes in tendons,^9^ is crucial for collagen fibril formation and interacts with type I and II collagens.^10^ Knockout studies have shown that a lack of type VI collagen results in reduced tendon stiffness and impaired mechanical signal transmission in the cartilage,^9^ emphasizing its role in maintaining the mechanical properties of tissues such as the TMJ disc, TMJ cartilage, and PDL, which face high occlusal forces. Thus, type VI collagen is vital in the oral tissues since it regulates fibrillogenesis and enhances mechanical resilience against occlusal pressure.

SPARC is the second most abundant ECM component in the TMJ disc tissue, following COL1 (Fig. 1a), and is highly expressed in the PDL tissue, reported in our previous study.^7^ It plays a key role in pro-collagen processing and collagen cross-linking by interacting with types I, III, IV, and V collagen.^11^ Its substantial expression is a hallmark of non-calcified connective tissues, such as the TMJ disc and PDL, with the cushioning and mechanical properties of SPARC maintained through its interaction with collagens. Among the proteoglycans (Fig. 1d), biglycan and fibromodulin were predominant in the TMJ discs. Studies have shown that double knockout of both biglycan and fibromodulin in mice leads to early degradation of the mandibular cartilage,^12^ highlighting their importance in fibrocartilaginous tissues. Biglycan and fibromodulin interact with collagen types I, II, and VI and regulate collagen fibrillogenesis.^13^ Therefore, high biglycan and fibromodulin expression may be a defining feature of the TMJ disc, contributing to its structural integrity by facilitating its connections with collagen. The interplay likely plays a crucial role in stabilizing the structural framework of the TMJ disc.

TMJ-derived cells lost some essential ECM components, such as type II collagen, decorin, and fibromodulin (Table 1), thereby affecting tissue functionality. Previous studies have shown that mechanical stimulation, such as compressive forces, can increase type II collagen expression in TMJ-derived cells^14^ while stretching enhanced decorin in the PDL.^15^ Decorin and fibromodulin are critical for collagen fibril formation, affecting fibril diameter and contributing to tissue structure.^13^ Therefore, replenishment of the lost ECM genes, such as type II collagen, decorin, and fibromodulin, along with collagen types I and VI, would be necessary for disc regeneration using cultured TMJ-derived cells.

## 5 Conclusion

In our study, collagen types VI and II were found to be prominently expressed, followed by collagen types I and III, in the TMJ disc tissues, reflecting the unique functions of the disc. Type VI collagen was highly expressed in both TMJ disc and PDL tissues. Thus, type VI collagen might be a key molecule for TMJ disc regeneration, providing elasticity and cushioning, and presenting new insights into TMJ regeneration.

### Patient's/Guardian's consent

This research did not involve human patients but rather animals; therefore, we obtained certification for animal experimentation from the Animal Care and Use Committee of Health Sciences University of Hokkaido, in accordance with the ARRIVE guidelines (Permission No. 22–027).

## Ethical approval

The animal experiments conducted in this study strictly followed the guidelines of the Animal Care and Use Committee of Health Sciences University of Hokkaido (permission NO. 22–027).

## Sources of funding

This study was partially supported by a Grant-in-Aid for the 2018–2019 Research Project from the Research Institute of Health Sciences at the Health Sciences University of Hokkaido, awarded to Toshiya Arakawa.

## Declaration of competing interest

The authors declare that there are no conflicts of interest.

## Figures and Tables

**Table:** Appendix Table. Primer Sequences for qPCR. All the primers for qPCR were designed using Primer-BLAST (https://www.ncbi.nlm.nih.gov/tools/primer-blast).

Gene	Primer	Sequence (5’–>3′)	Amplicon (bp)	Accession Number
COL1A1	Forward	CTGACGCATGGCCAAGAAGA	88	XM_032912698.1
	Reverse	CATCGGGTTTCCACGTCTCA		
COL2A1	Forward	CGCTCAAGTCGCTGAACAAC	233	NM_012929.1
	Reverse	GTTCTTCCGAGGCACAGTCG		
COL6A2	Forward	TGGAAGACGTCCTTTGTCCG	180	NM_001100741.1
	Reverse	GTGACACCTCCTCCACGAAG		
Beta-actin	Forward	ACCCGCGAGTACAACCTTC	75	NM_031144.3
	Reverse	ATCGTCATCCATGGCGAACTG		

**Figure:**
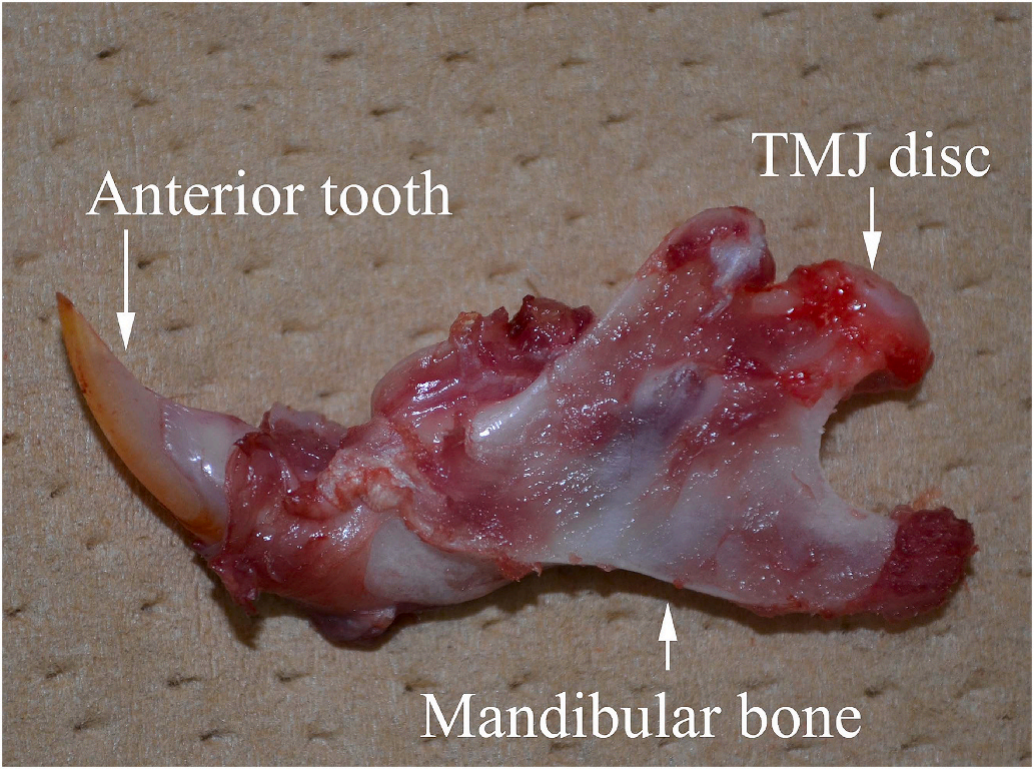
The surgical procedure performed on a rat, where the TMJ disc was attached to the mandibular condyle.

**Figure:**
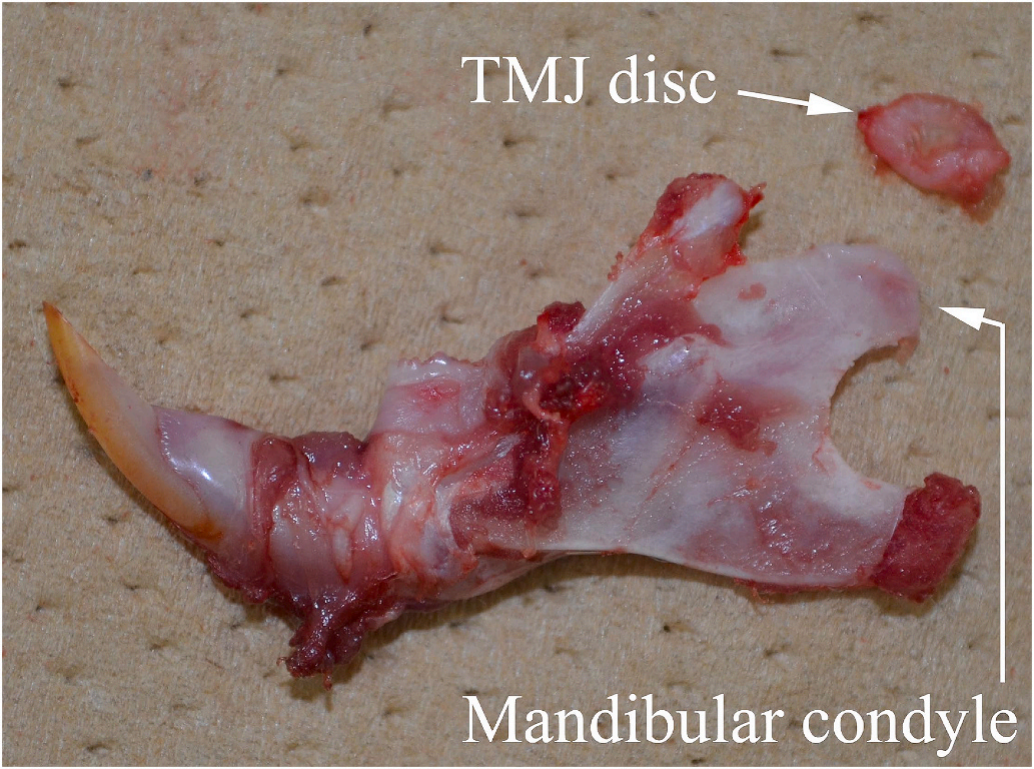
The surgical procedure performed on a rat, where the TMJ disc was extracted from the mandibular condyle.
